# The Relationship between Renal Resistive Index and Complexity of Coronary Lesions in Patients with Stable Coronary Artery Diseases

**DOI:** 10.31083/j.rcm2505160

**Published:** 2024-05-09

**Authors:** Hesham Refaat, Ayman Tantawy

**Affiliations:** ^1^Cardiology Department, Zagazig University, 44519 Zagazig, Egypt

**Keywords:** coronary artery disease, renal resistive index, SYNTAX score

## Abstract

**Background:**

The most common cause of coronary artery diseases (CAD) is atherosclerosis. The synergy between percutaneous coronary intervention with TAXUS™ and cardiac surgery (SYNTAX) score was used to assess complex CAD lesions. The renal resistive index (RRI) is a Doppler ultrasound parameter calculated to assess renal haemodynamics. The direct relationship between CAD complexity and RRI was not yet investigated. The aim of our study was to investigate this relationship between RRI and SYNTAX score in stable CAD patients.

**Methods:**

This study included 214 patients with stable CAD and subsequent coronary angiography done at our institution. Regarding CAD complexity, these patients were classified into 166 patients with low SYNTAX score (SYNTAX ≤22), and 48 patients with high SYNTAX score (SYNTAX >22). The demographic, laboratory, clinical, echocardiographic data and renal Doppler parameters; including RRI, were recorded.

**Results:**

Multivariate logistic regression analysis demonstrated that RRI (odds ratio, OR = 4.440, 95% (confidence interval) CI: 1.418–13.903, *p* = 0.010) was a novel independent predictor of high SYNTAX score in patients with stable CAD, in addition to other traditional predictors as diabetes mellitus (OR = 4.401, 95% CI: 1.081–17.923, *p* = 0.04), low-density lipoprotein cholesterol (LDL-C) (OR = 2.957, 95% CI: 1.920–8.995, *p* = 0.027), multi-vessel CAD (OR = 2.113, 95% CI: 1.241–2.280, *p* = 0.001) and Gensini score (OR = 6.539, 95% CI: 1.977–21.626, *p* = 0.002). Receiver operator characteristic curve analysis showed that RRI >0.655 (sensitivity of 80%, specificity of 73.6%) was the best cut-off value for predicting high SYNTAX score.

**Conclusions:**

The non-invasively measured RRI is closely associated with high SYNTAX score in stable CAD patients.

## 1. Introduction

Cardiovascular diseases (CVD) are the commonest cause of morbidity and mortality worldwide. The commonest underlying mechanism of coronary artery diseases (CAD) is atherosclerosis. Atherosclerotic plaques develop over years leading to a long-term clinically silent coronary obstruction, up to sudden plaque rupture or erosion with subsequent development of acute coronary syndrome (ACS) [1].

Chronic coronary syndromes (CCS), unlike ST-segment elevation myocardial infarction (STEMI), have different predictors of long-term outcomes [2] including left ventricular ejection fraction (LVEF), age, complete revascularization [3], associated acute left ventricular failure, peak troponin level and degree of ST-segment deviation [4]. Also, chronic kidney diseases (CKD) are increasingly becoming a key predictor of cardiac morbidity and mortality [5].

The synergy between percutaneous coronary intervention with TAXUS™ and cardiac surgery (SYNTAX) score was calculated to evaluate both CAD extent and complexity. Moreover, this score could be considered as a strong predictor of major adverse cardiac events (MACE) [6] and cardiovascular death in patients subjected to percutaneous coronary intervention (PCI) [7]. Each coronary lesion could be scored separately using SYNTAX scoring system, and then the total SYNTAX score is achieved by summing all these calculated scores.

The cardio-renal syndrome is the coexistence of both cardiac and renal pathology, and is related to both humoral and neural signaling [8]. Analysis of micro- and macrovascular circulatory parameters could be used for early detection of CVD and related vascular damage. Among these parameters, it was found that renal resistive index (RRI) could be considered as the most reproducible and clinically relevant index of renal hemodynamics and vascular stiffness [9].

RRI is a simple non-invasive renal Doppler Ultrasonography (USG) parameter reflecting renal haemodynamic changes and allowing assessment of renal vascular damage and resistance [10]. Also, RRI was dependent on numerous factors including renal vascular stiffness, age, brady- and tachyarrhythmias, pulse pressure, significant valvular lesions and pathological lesions within renal parenchyma [11].

High RRI was found to be correlated with impaired renal haemodynamics [12]. This could be used to allow accurate prediction of the onset of acute kidney injury and its persistence [13], arterial remodeling and stiffness [9], and adverse CVD events in elderly patients especially those having a hypertensive renal disease [14]. Moreover, in a large cohort enrolling CKD patients, high RRI was significantly associated with increased mortality [15].

Of note, to our knowledge, none of previously published studies assessed the relationship between RRI and CAD. In our study, the aim was to prove the hypothesis that using intra-renal flow parameters may define complex CAD in stable CAD patients by evaluating the association between RRI and extent and complexity of CAD, defined by SYNTAX score.

## 2. Methods

### 2.1 Study Population

Between February 2021 and August 2022, a total of 254 consecutive patients with the inclusion criteria of CCS [16] and a clinical indication for coronary angiogram based on former non-invasive stress tests, have been included in this cross-sectional study with subsequent coronary angiography done at our hospital.

Patients with pulmonary edema, cardiogenic shock, any type of respiratory failure, previous coronary artery bypass grafting (CABG), CKD defined as proteinuria level >500 mg/L or estimated glomerular filtration rate (eGFR) <50 mL/min/1.73 $m^{2}$, renal artery stenosis, malignancy, nephrectomy, haemorrhagic diathesis, infective or inflammatory diseases, and suspected pregnancy were excluded (n = 27 patients). Additionally, the exclusion criteria included other conditions affecting intra-renal hemodynamic parameters; including moderately severe valvular aortic stenosis, other severe valvular lesions, tachyarrhythmia >100 bpm or bradyarrhythmia <50 bpm, high pulse pressure >80 mmHg (n = 13 patients).

Finally, 214 patients were enrolled and classified according to the CAD severity; revealed by coronary angiogram and assessed by SYNTAX score, into two main groups: group (1) including 166 patients with low SYNTAX score (SYNTAX ≤22), and group (2) including 48 patients with high SYNTAX score (SYNTAX >22). The study was carried out in adherence to the principles of the Declaration of Helsinki on Biomedical Research Involving Human Subjects. The Ethics Committee of Zagazig university approved the study protocol (ZU-IRB #62/5). All study population already gave written informed consents prior to study participation.

### 2.2 Clinical Data Collection 

Baseline demographics and clinical characteristics of all included patients were obtained from our hospital records. Cardiovascular risk factors of all patients were identified. The diagnostic criteria of diabetes mellitus were confirmed in all patients receiving active treatment or having an abnormal fasting glucose (>126 mg/dL) or abnormal 2 h postprandial levels (>200 mg/dL). Hypertensive patients were identified as those taking antihypertensive therapy or those with systolic blood pressure (SBP) >140 mmHg and/or a diastolic blood pressure (DBP) >90 mmHg. Dyslipidemia was diagnosed with a total cholesterol level >200 mg/dL or low-density lipoprotein cholesterol (LDL-C) >100 mg/dL, or when the patients were already on lipid-lowering therapy according to Adult Treatment Panel III Guidelines [17]. Family history of premature CAD was defined as the presence of CAD in the first-degree relatives before 55 years old for men and 65 years old for women. Patients using tobacco products and those with smoking cessation within one month could be considered smokers [18].

### 2.3 Blood Samples and Laboratory Analysis 

Venous blood samples were withdrawn from all patients on admission prior to coronary angiography. The following laboratory parameters were obtained from all patients: complete blood count, lipid panel, serum albumin level, and creatinine level. The eGFR was measured according to the abbreviated Modification of Diet in Renal Disease (aMDRD) equation: aMDRD = 186 × (serum creatinine, mg/dL) – 1.154 × (age) – 0.203 × 0.742 (if female) × 1.210 (if African) [19].

### 2.4 Electrocardiogram (ECG) Analysis and Echocardiography Protocol

A resting ECG was obtained all patients on admission. Transthoracic echocardiography (TTE) was done before coronary angiography by an experienced blinded cardiologist according to the current practice guidelines [20] to assess the diastolic functions and LVEF by the modified Simpson’s rule.

### 2.5 Coronary Angiography Protocol 

Coronary angiography was done using Siemens (Axiom Sensis XP, Berlin, Germany) device at our catheterization laboratory via either radial or femoral access using 6 or 7 Fr sheaths and catheters with administrating 0.2 mg of intracoronary nitroglycerin. Multiple projections were used for adequate analysis of target lesions characteristics by two experienced blinded interventional cardiologists.

### 2.6 Calculation of SYNTAX and GensiniScores

According to lesion classification system based on the American College of Cardiology/American Heart Association (ACC/AHA) [21], patients with left main coronary artery (LMCA) stenosis >50% and/or other epicardial coronary arteries stenosis >70% were defined as obstructive coronary lesions. The SYNTAX score was calculated for each patient by a blinded interventional cardiologist using the online SYNTAX score calculator system (http://www.syntaxscore.com) [22]. The severity of CAD was also classified according to the Gensini score; to score both extent and degree of coronary artery stenosis [23].

### 2.7 Renal USG and Doppler Protocols

All included patients underwent the renal ultrasound study using a 5C probe (4.4–6.7 MHz). The Doppler analysis of blood flow indices within arcuate or interlobular renal arteries was done by an experienced operator. After 6 hours of fasting, the patients were evaluated after resting for at least 20 minutesin supine position and the following parameters were assessed: (a) the morphology of kidneys to obtain their length, width, parenchymal thickness and potential structural abnormalities requiring further evaluation, (b) renal artery hemodynamics screening using peak systolic velocity (PSV) and end-diastolic flow velocity (EDV) within the main renal artery, and (c) mean velocity (MV), acceleration time (AT), augmentation index (AI) of intra-renal arteries using a 2–4 mm pulsed wave Doppler.

These mentioned Doppler parameters were used to calculate both RRI and renal pulsatility index (RPI) based on these formulas: RRI = (PSV – EDV)/PSV and RPI = (PSV – EDV)/MV. These indices were obtained 3 times involving the interlobular renal arteries located in upper, mid and lower kidney poles using a pulsed-wave Doppler with the calculation of arithmetic means of these measurements [24].

## 3. Statistical Analyses

Data distribution was first assessed using the Kolgormonov-Smirnov test. Then, categorical data were compared based on the chi-square test or Fisher exact test. Continuous variables were also compared according to an unpaired Student’s *t*-test or Mann–Whitney U-test. Data were expressed as mean ± standard deviation. The independent predictors of high SYNTAX score in those patients having stable CAD were assessed using the multivariate analysis; including all significant variables based on the *p*-value of the univariate regression analyses (*p* < 0.05). Receiver operating characteristic curve (ROC) was performed to detect the best cut off value of RRI for defining high SYNTAX score. Two-sided statistical tests were achieved with *p*-value of <0.05 representing a statistically significant difference. All these analyses were done using SPSS version 20 (SPSS Inc, Armonk, NY, USA).

## 4. Results

A total of 214 patients with stable CAD underwent coronary angiography at our institution were enrolled in the present study. Subsequently, out of these 214 patients, 166 patients had low SYNTAX score (SYNTAX ≤22) (77.6%) and the remaining 48 patients had high SYNTAX score (SYNTAX >22) (22.4%).

### 4.1 Demographical and Clinical Characteristics

Baseline demographic data and clinical characteristics are shown in Table 1. This study population included 142 males (66.4%) and 72 females (33.6%). Out of high SYNTAX score group, 62.5% were males compared to 67.5% in low SYNTAX score group (*p* = 0.52). Similarly, no differences were significantly noted between two groups regarding the mean age of patients (*p* = 0.97).

**Table 1.** **Demographic, clinical, and laboratory characteristics of overall population, according to SYNTAX classification**.

Variable			All Patients	SYNTAX ≤22	SYNTAX >22	*p* value
			(n = 214)	(n = 166)	(n = 48)	
Clinical characteristics						
	Age, mean ± SD, years		64.02 ± 11.84	64.04 ± 12.28	63.96 ± 10.15	0.97
	Male sex, n (%)		142 (66.4%)	112 (67.5%)	30 (62.5%)	0.52
Cardiovascular risk factors, n						
	Hypertension		174 (81.3%)	138 (83.1%)	36 (75.0%)	0.20
	Smoking		104 (48.6%)	73 (44.0%)	31 (64.6%)	0.01
	Dyslipidemia		104 (48.6%)	73 (44.0%)	31 (64.6%)	0.01
	Diabetes Mellitus		100 (46.7%)	69 (41.6%)	31 (64.6%)	0.005
	Family history of CAD		80 (37.4%)	58 (34.9%)	22 (45.8%)	0.17
	Obesity		36 (16.8%)	26 (15.7%)	10 (20.8%)	0.39
	BMI (kg/$m^{2}$)		26.1 ± 2.70	25 ± 2.60	27 ± 2.70	0.27
	SBP, mmHg		131.59 ± 30.76	126.15 ± 27.74	133.16 ± 31.48	0.16
	DBP, mmHg		72.43 ± 17.41	67.73 ± 17.24	73.80 ± 1.28	0.03
	HR, bpm		87.64 ± 18.36	84.02 ± 18.99	88.69 ± 18.01	0.12
	LVEF, %		55.00 ± 6.45	58.20 ± 4.97	51.80 ± 6.57	0.12
LV diastolic dysfunction						
	Grade 1		98 (45.8%)	82 (49.4%)	16 (33.3%)	
	Grade 2		90 (42.1%)	67 (40.4%)	23 (47.9%)	0.09
	Grade 3		26 (12.1%)	17 (10.2%)	9 (18.8%)	
Medications						
	Beta-blockers		210 (98.1%)	164 (98.8%)	46 (95.8%)	0.18
		CCBs	204 (95.3%)	158 (95.2%)	46 (95.8%)	0.85
		Nitrates	168 (78.5%)	134 (80.7%)	34 (70.8%)	0.14
		Aspirin	166 (77.6%)	126 (75.9%)	40 (83.3%)	0.28
		ACEIs/ARBs	24 (11.2%)	22 (13.3%)	2 (4.2%)	0.08
	Statins		172 (80.4%)	130 (78.3%)	42 (87.5%)	0.16
Laboratory characteristics						
	Hemoglobin, gm/dL		12.84 ± 1.05	13.08 ± 0.94	12.60 ± 1.11	0.5
	Leukocytes (×${\mathrm{10}}^{3}$), µL		8.14 ± 1.49	7.56 ± 1.31	8.72 ± 1.56	0.24
	Platelet count (×${\mathrm{10}}^{3}$), µL		226.50 ± 74.01	225.18 ± 73.43	231.08 ± 76.63	0.63
	TGs, mg/mL		155.02 ± 55.59	156.14 ± 58.77	151.13 ± 43.19	0.58
	LDL-C, mg/dL		129.08 ± 31.89	122.55 ± 24.44	151.64 ± 42.94	<0.001
	HDL-C, mg/dL		38.86 ± 7.61	38.91 ± 7.56	38.65 ± 7.87	0.83
	Serum albumin, g/L		35.73 ± 3.39	35.56 ± 3.32	36.28 ± 3.63	0.11
	Creatinine, mg/dL		1.02 ± 0.47	1.03 ± 0.48	1.01 ± 0.47	0.82
	eGFR, mL/min/1.73 $m^{2}$		94.01 ± 42.37	91.37 ± 40.41	103.17 ± 47.89	0.13

ACEIs, angiotensin-converting enzyme inhibitors; ARBs, angiotensin receptor blockers; bpm, beats per minutes; CAD, coronary artery disease; CCBs, calcium channel blockers; DBP, diastolic blood pressure; eGFR, estimated glomerular filtration rate; HDL-C, high density lipoprotein cholesterol; HR, heart rate; LDL-C, low density lipoprotein cholesterol; LV, left ventricle; LVEF, left ventricular ejection fraction; SBP, systolic blood pressure; TGs, triglycerides; BMI, body mass index; SYNTAX, percutaneous coronary intervention with TAXUS™ and cardiac surgery.

Compared to those with low SYNTAX scores, diabetes mellitus, dyslipidemia and smoking habit were more frequent in high SYNTAX scores patients (64.6% *vs*. 41.6%, *p* = 0.005 & 64.6% *vs*. 44.0%, *p* = 0.01 & 64.6% *vs*. 44.0%, *p* = 0.01, respectively). No significant differences were noticed regarding other demographic and clinical data between these two groups.

### 4.2 Laboratory Characteristics

Laboratory features are shown in Table 1. Compared to low SYNTAX score group, it was noticed that LDL-C level was significantly higher in patients included in high SYNTAX score group (151.64 ± 42.94 *vs*. 122.55 ± 24.44 mg/dL, *p* < 0.001), with no significant difference regarding triglycerides (TGs) (*p* = 0.58) and HDL-C (*p* = 0.83) levels. Regarding other laboratory parameters, there weren’t significant differences between both groups.

### 4.3 Angiographic Characteristics 

Angiographic features are shown in Table 2. The mean SYNTAX score value was 15.16 ± 8.05 in all patients, whereas those included in low SYNTAX score group had a mean score of 11.39 ± 3.98 compared to 28.21 ± 3.78 in the high SYNTAX score group (*p* < 0.001). Concurrently, the mean Gensini score was 41.34 ± 33.21 in all included patients, and it was significantly higher in those with a higher SYNTAX score than others with a lower SYNTAX score (99.79 ± 19.71 *vs*. 24.44 ± 5.71, *p* < 0.001).

**Table 2.** **Angiographic characteristics of overall population, according to SYNTAX classification**.

Variable	All Patients	SYNTAX ≤22	SYNTAX >22	*p* value
	(n = 214)	(n = 166)	(n = 48)	
Angiographic characteristics				
Multi-vessel CAD	101 (47.2%)	71 (42.8%)	30 (62.5%)	0.02
Left main CAD	26 (12.1%)	14 (8.4%)	12 (25.0%)	0.002
SYNTAX score	15.16 ± 8.05	11.39 ± 3.98	28.21 ± 3.78	<0.001
Gensini score	41.34 ± 33.21	24.44 ± 5.71	99.79 ± 19.71	<0.001

CAD, coronary artery disease; SYNTAX, percutaneous coronary intervention with TAXUS™ and cardiac surgery.

Of note, multi-vessel CAD was noted in 47.2% of all included patients, and it was more frequent in those with a higher SYNTAX score than those with a lower SYNTAX score (62.5% *vs*. 42.8%, *p* = 0.02). Similarly, left main CAD was noticed in 12.1% and its prevalence is significantly higher in high SYNTAX score group compared to that with low SYNTAX score (25.0% *vs*. 8.4%, *p* = 0.002).

### 4.4 Renal Doppler USG Characteristics 

Renal USG and Doppler characteristics are shown in Table 3. Regarding USG characteristics, the mean values were 44.08 ± 3.01 mm for renal width, 104.22 ± 5.51 mm for renal length, and 14.51 ± 1.33 mm for renal parenchymal thickness, without significant differences between both groups regarding these parameters (*p* = 0.91, *p* = 0.58, and *p =* 0.95, respectively).

**Table 3.** **Renal USG characteristics of overall population, according to SYNTAX classification**.

Variable	All Patients	SYNTAX ≤22	SYNTAX >22	*p* value
	(n = 214)	(n = 166)	(n = 48)	
Renal USG characteristics				
Renal width, mm	44.08 ± 3.01	43.96 ± 2.14	44.20 ± 4.12	0.91
Renal length, mm	104.22 ± 5.51	103.18 ± 4.79	105.26 ± 6.52	0.58
Renal parenchymal thickness, mm	14.51 ± 1.33	14.48 ± 1.22	14.54 ± 1.57	0.95
Aorta PS, cm/s	65.57 ± 23.64	66.96 ± 24.97	59.51 ± 15.78	0.12
PSV, m/s	57.46 ± 27.28	57.48 ± 27.47	57.36 ± 27.17	0.99
RPI	1.37 ± 0.40	1.38 ± 0.41	1.34 ± 0.38	0.79
RRI	0.632 ± 0.076	0.614 ± 0.072	0.699 ± 0.047	<0.001

PS, peak systolic; PSV, peak systolic velocity; RPI, renal pulsatile index; RRI, renal resisitive index; USG, ultrasonography; SYNTAX, percutaneous coronary intervention with TAXUS™ and cardiac surgery.

Regarding renal Doppler characteristics, there were no significant differences between two SYNTAX groups regarding mean values of PSV (57.48 ± 27.47 *vs*. 57.36 ± 27.17 m/s, *p* = 0.99) and RPI (1.38 ± 0.41 *vs*. 1.34 ± 0.38, *p* = 0.79). However, in comparison with low SYNTAX score group, mean RRI value was significantly higher in patients included in the high SYNTAX score group (0.699 ± 0.047 *vs*. 0.614 ± 0.072, *p* < 0.001).

**Fig. 1.**
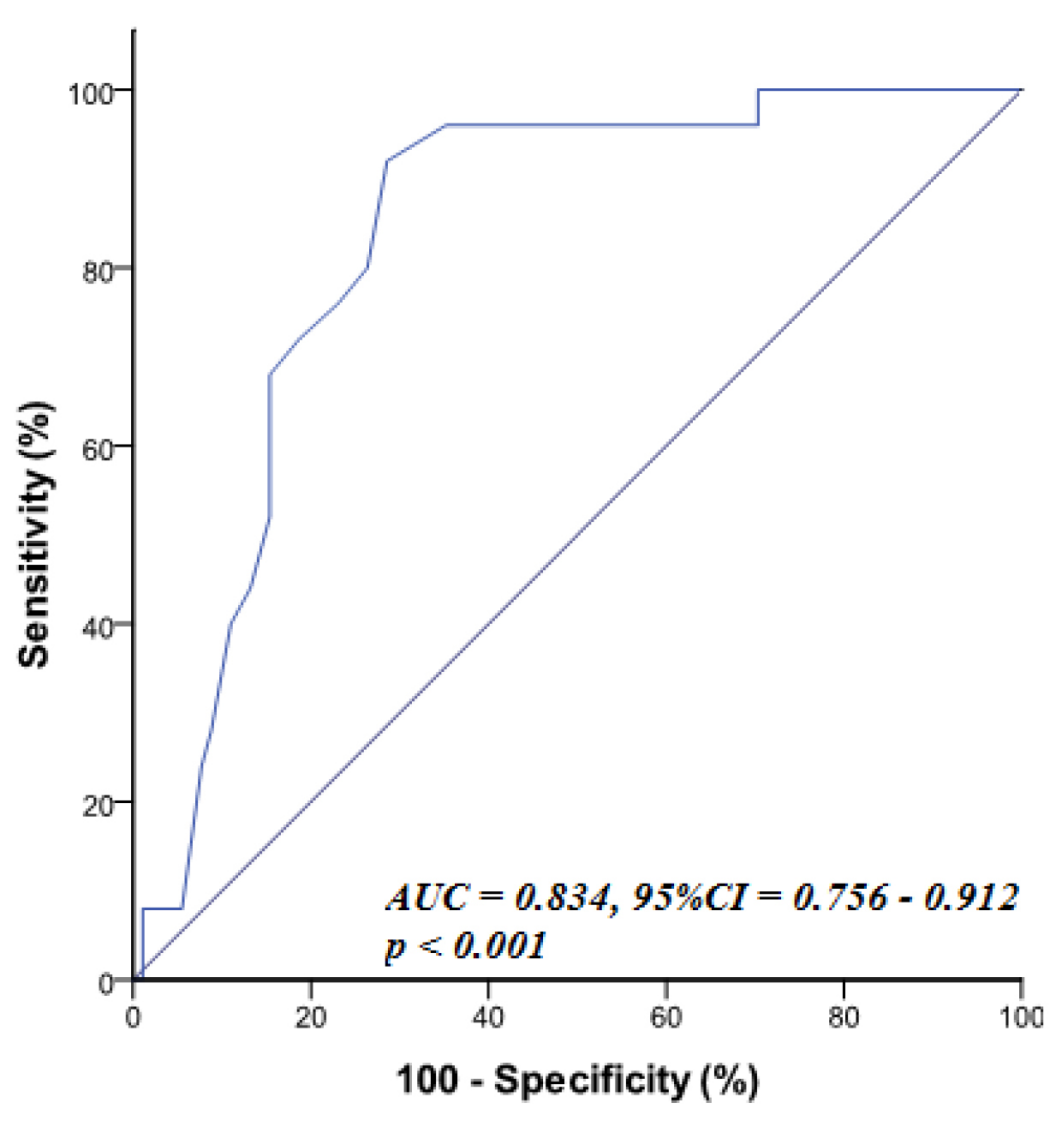
**ROC curve of RRI for prediction of high syntax score (SYNTAX >22)**. RRI >0.655 (sensitivity of 80%, specificity of 73.6%). SYNTAX, percutaneous coronary intervention with TAXUS™ and cardiac surgery; AUC, area under curve; RRI, renal resistive index; ROC, receiver operating curve.

ROC statistical analyses (Table 4) showed that RRI >0.655 (sensitivity of 80%, specificity of 73.6%) was the best cut-off value for predicting high SYNTAX score (SYNTAX >22) in patients with stable CAD (Fig. 1).

**Table 4.** **Sensitivity and specificity for RRI to predict high SYNTAX score (SYNTAX >22) in patients with stable CAD**.

Variable	AUC	*p*-value	95% CI	Cut off	Sensitivity	Specificity
RRI	0.834	<0.001	0.756–0.912	> 0.655	80.0%	73.6%

RRI, renal resistive index; SYNTAX, percutaneous coronary intervention with TAXUS™ and cardiac surgery; CAD, coronary artery disease; AUC, area under curve.

### 4.5 Independent Predictors of High SYNTAX Score

The independent predictors of complex CAD in stable CAD patients; defined by SYNTAX score >22 were summarized in Table 5. In univariate logistic regression analysis, significant relations were noticed between diabetes mellitus (*p* = 0.005), smoking (*p* = 0.01), LDL-C level (*p* < 0.001), multi-vessel CAD (*p* = 0.02), LMCA lesions (*p* = 0.002), Gensini score (*p* < 0.001) and RRI (*p* < 0.001) with a higher incidence of SYNTAX score >22 in patients with stable CAD.

**Table 5.** **Univariate and multivariate regression analysis for the parameters affecting high SYNTAX score (SYNTAX >22) in patients with stable CAD**.

Variables	Univariate		Multivariate	
	*p*-value	OR	*p*-value	OR
		(95% CI)		(95% CI)
Diabetes mellitus	0.005	2.564	0.040	4.401
		(1.315–4.996)		(1.081–17.923)
Smoking	0.01	2.323	0.087	2.717
		(1.193–4.523)		(0.799–27.847)
LDL-C, mg/dL	<0.001	1.035	0.027	2.957
		(1.020–1.050)		(1.920–8.995)
Multi-vessel CAD	0.02	2.230	0.001	2.113
		(1.152–4.316)		(1.241–2.280)
Left main CAD	0.002	3.619	0.279	0.874
		(1.543–8.487)		(0.527–3.176)
Gensini score	<0.001	3.073	0.002	6.539
		(1.944–4.859)		(1.977–21.626)
RRI	<0.001	1.751	0.010	4.440
		(3.628–8.450)		(1.418–13.903)

CAD, coronary artery disease; LDL-C, low density lipoprotein cholesterol; RRI, renal resistive index; OR, odds ratio; SYNTAX, percutaneous coronary intervention with TAXUS™ and cardiac surgery.

In the multivariate logistic regression analysis, diabetes mellitus (odds ratio, OR = 4.401, 95% CI: 1.081–17.923, *p* = 0.04), LDL-C (OR = 2.957, 95% CI: 1.920–8.995, *p* = 0.027), multi-vessel CAD (OR = 2.113, 95% CI: 1.241–2.280, *p* = 0.001), Gensini score (OR = 6.539, 95% CI: 1.977–21.626, *p* = 0.002), and RRI (OR = 4.440, 95% CI: 1.418–13.903, *p* = 0.010) were found to be strong independent predictors of a higher SYNTAX score in stable CAD patients.

## 5. Discussion

The main findings of our study were: (1) in stable CAD patients, diabetes mellitus, dyslipidemia, and smoking were more frequent in those with high SYNTAX score group (SYNTAX >22) compared to others with low SYNTAX score (SYNTAX ≤22), (2) Moreover, left main and multi-vessel CAD were more pronounced in high SYNTAX score patients (SYNTAX >22) along with higher Gensini score, compared to those with lower SYNTAX score (SYNTAX ≤22), (3) RRI, as determined by the non-invasive renal Doppler study, was significantly higher in the patients with more complex CAD than in those with lower CAD complexity, and (4) RRI along with diabetes mellitus, LDL-C levels, multivessel CAD and Gensini score could be considered as strong independent predictors of complex CAD with higher SYNTAX score (SYNTAX >22) in stable CAD patients.

Atherosclerosis is a chronic immune-inflammatory and fibro-proliferative disease with a systemic and progressive behaviour. It is heterogeneously distributed in coronary plaques and frequently affects peripheral vasculature as renal arteries in addition to aorta and coronary arteries. In coronary arteries, atherosclerosis is characterized by its fatty streaks in childhood. However, in adulthood, fibrous plaques are more noted with more complex and advanced coronary lesions [25]. Concurrently, the kidneys are characterized by their significant arterial vascular structure, and also are affected by atherosclerosis lie coronary arteries where it leads to both anatomical and functional effects in the kidneys [26].

Our study included 214 patients with stable CAD who had been admitted to our catheterization laboratory to perform coronary angiography. According to complexity of atherosclerotic coronary lesions defined by the SYNTAX score, these patients were subsequently divided into those with SYNTAX score ≤22 and others with a higher SYNTAX score >22. The role of old age, male gender, smoking habit, diabetes mellitus and obesity as major determinants of complex CAD lesions has been discussed extensively before, where the publication stated by the Framingham Heart study and a series of landmark studies reported a causal relationship with atherosclerosis [27].

Within the same context, the present study reported a higher frequency of smoking (64.6% *vs*. 44%, *p* = 0.01) and diabetes mellitus (64.6% *vs*. 41.6%, *p* = 0.005) among patients with stable CAD and having complex coronary lesions (SYNTAX >22) compared to those with less severe coronary lesions (SYNTAX ≤22). This could be attributed to the fact that smoking affects atherosclerosis phases; from endothelial dysfunction to adverse clinical events [28]. Also, in diabetics, it was stated that hyperglycemia, insulin resistance and free fatty acid release could lead to a significantly increased oxidative stress, and therefore atherosclerosis acceleration [29]. Moreover, dyslipidemia was more frequent in stable CAD patients included in this study with a higher SYNTAX score.

RRI is a non-invasive renal Doppler modality used to assess renal hemodynamics. Moreover, RRI could be considered as a result of complex haemodynamic interactions involving both the kidneys and the systemic vasculature, and most of those interactions are not entirely understood yet. Kintis* et al*. [30] stated that RRI provides also a prognostic information regarding the systemic vasculature and could be considered as a promising marker for vascular damage, where high RRI could result in adverse clinical outcomes, especially in elderly, hypertensive, and diabetic patients.

This strong association between elevated RRI and systemic vascular damage was reported in many other previous studies where Prejbisz *et al*. [31] reported that a higher RRI was noticed in patients having a truly resistant hypertension than those with a well controlled systemic hypertension (0.62 ± 0.05 *vs*. 0.60 ± 0.05, *p* < 0.05). Also, RRI revealed a significant correlation with both pulse and ambulatory blood pressure values, fasting glucose levels and E/e’ ratio [31]. It was noted that the metabolic effects of RRI could be illustrated in patients with type 2 diabetes mellitus, where dynamic values; RRI changes after sublingual nitrate, could predict the microalbuminuria onset [32]. Also, Geraci *et al*. [33] stated that RRI values were greatly correlated with both the extent of carotid atherosclerosis and carotid intima-media thickness (IMT) assessed in hypertensive patients.

More recently, Geraci *et al*. [34] reported that the Doppler-based intra-renal flow parameters were greatly associated with severity of atherosclerotic lesions assessed in coronary angiography, but only noted among those patients having less pronounced atherosclerosis. Of note, this significant correlation was found to be valid for RPI, but not RRI, which needs further explanations. On this basis, there are no sufficient studies discussing the correlation between extent and severity of atherosclerotic CAD and RRI, and thus, we hypothesized in this study that RRI values determined by renal Doppler studies could be used to investigate renal vasculature and correlating it with the atherosclerotic CAD severity to assess the RRI value to predict its progression.

Accordingly, the present study shed light on the relationship between RRI and the complex coronary lesions in patients with stable atherosclerotic CAD. It was also found that RRI was higher in patients having more complex coronary lesions (SYNTAX >22) than those having less severe coronary lesions (SYNTAX ≤22) (0.699 ± 0.047 *vs*. 0.614 ± 0.072, *p* < 0.001). This finding was in accordance with Higuchi *et al*. [35] that reported a significant correlation between higher RRI and patients having multi-vessel diseases than those with only single or double vessel diseases (*p* < 0.01). Within the same context, Alan *et al*. [36] reported that RRI values were also higher in CAD patients with severe lesions compared to those with mild lesions (*p* < 0.001).

However, our results came in contrast with Demirtaş and Bulut who reported that RRI in the low-risk ACS patients (mean SYNTAX Score <12) was 0.73 ± 0.07 while in high risk patients (mean SYNTAX Score ≥12) was 0.76 ± 0.08 without significant differences between both groups. Higher RRI observed in this high risk group may be related to acute stress, however, non-significant difference could be attributed to difficult Doppler assessment of the kidneys due to retroperitoneal location, difficult assessment in obese patients and limited efficacy of shear wave elastography (SWE) using the acoustic radiation force impulse imaging modality in the kidneys located deeper than 5 cm [37]. Of note, these results could be considered as novel ones as the study exclusively included stable CAD patients; not ACS patients, without renal impairment (eGFR ≥50 mL/min/1.73 $m^{2}$). Thus, the current study highlights the valuable clinical impacts of RRI regardless of the kidney status, which could greatly affect the RRI results.

The present study has a great clinical implication where it documents that RRI could be considered as a novel strong independent predictor of high SYNTAX score in stable atherosclerotic CAD, besides other traditional predictors as diabetes mellitus, LDL-C level, multi-vessel CAD, and Gensini score. In line with these findings, the cohort study stated by Pearce *et al*. [14] revealed a significant association between Doppler renal parameters; including RRI and RPI, and adverse cardiovascular events. Also, Akgul *et al*. [38] proved a relationship between RRI and both cardiac risk factors and carotid IMT. It was stated that a mild renal dysfunction has been also reported to be a strong independent risk factor for cardiovascular mortality, even after proper controlling of other atherosclerotic risk factors [39].

The main underlying mechanisms illustrating the higher SYNTAX score associated with high RRI values are still uncertain. However, this could be attributed to a histological study showing that the reno-vascular atherosclerosis is considered as independent risk factor for an increased RRI. Moreover, atherosclerosis is a systemic process, and its main pathophysiology is nearly the same in all involved patients [40]. Also, recent studies including hypertensive patients documented a significant correlation between arterial stiffness index [41], central pulse pressure, aortic stiffness [42] and RRI. Accordingly, the RRI could be a novel indicator of a systemic vascular injury rather than only a renal vascular damage. Moreover, Wybraniec *et al*. [24] stated that the mechanism underlying this predictive role of RRI could be attributed to its correlation with both vascular remodelling (stiffness) and vascular sympathetic tone (renal artery constriction). It was stated that a higher sympathetic tone within renal arcuate and interlobular arteries could lead to both lower EDV and higher RRI values in high risk atherosclerotic CAD patients [24]. The present study has a unique design as it compensated for intrarenal flow variations by excluding those patients having valvular heart disease that could be considered as a significant determinant of RRI and also adverse long-term outcomes.

Alan *et al*. [36] analysed the diagnostic clinical performance of RRI values in ROC analyses to differentiate CAD severity according to Gensini score. They found that the best cut-off value was 0.605 with sensitivity of 80.60% and specificity of 66.70% [36]. Furthermore, Calabia *et al*. [43] found high specificity and sensitivity values for RRI in determining arterial vascular stiffness. In this study, the best cut-off RRI value to differentiate patients with a high SYNTAX score was >0.655 with sensitivity of 80% and specificity of 73.6%. Accordingly, we suggest that RRI values determined by renal Doppler studies may provide useful information about the progression of CAD before being irreversible with a high degree of complexity, and thus allowing early treatment of CAD. These findings were supported by Rosławiecka *et al*. [44] who stated that patients having renal artery stenosis with a concomitant atherosclerotic lesion (>30% stenosis) in the contralateral renal artery are associated with adverse cardiovascular events.

## 6. Limitations

The present study had some limitations. First, it is a single-centre study including small number of certain patients with stable CAD. Thus, multicentre studies including more patients could have more significant data and results to investigate the correlation between increased RRI and cardiovascular mortality and adverse events. Second, RRI is limited by its dependence on numerous variables altering the intra-renal hemodynamics. However, patients with severe valvular lesions of any kind, high pulse pressure, tachy- and bradyarrhythmias were excluded from this study. Third, this study didn’t evaluate the potential effects of drugs taken by some patients prior to RRI assessment. Lastly, the measurements of Doppler parameters as PSV and EDV and their derivatives are influenced by inter- and intra-observer variability. Thus, measurements were repeated in kidneys using the arithmetic mean of these measurements.

## 7. Conclusions

Atherosclerosis is commonly associated with the affection of both coronary and renal vasculatures. In patients with stable atherosclerotic CAD, the RRI is significantly associated with both angiographic extent and complexity of CAD; and not only a specific marker for renal vascular damage. Thus, this RRI could be considered as a diagnostic modality which is easily accessible to improve the stratification of cardiovascular risk and provide additional prognostic information in stable CAD patients referred to invasive procedures.

## Data Availability

The datasets used and/or analyzed during the current study are available from the corresponding author on reasonable request.
